# Impact of thyroid autoimmunity and vitamin D on *in vitro* fertilization/intracytoplasmic sperm injection outcomes among women with normal thyroid function

**DOI:** 10.3389/fendo.2023.1098975

**Published:** 2023-05-08

**Authors:** Yalong Liu, Zining He, Ning Huang, Lin Zeng, Yang Wang, Rong Li, Hongbin Chi

**Affiliations:** ^1^ Center for Reproductive Medicine, Department of Obstetrics and Gynecology, Peking University Third Hospital, Beijing, China; ^2^ National Clinical Research Center for Obstetrics and Gynecology (Peking University Third Hospital), Beijing, China; ^3^ Key Laboratory of Assisted Reproduction (Peking University), Ministry of Education, Beijing, China; ^4^ Beijing Key Laboratory of Reproductive Endocrinology and Assisted Reproductive Technology, Peking University Third Hospital, Beijing, China

**Keywords:** *in vitro* fertilization, intracytoplasmic sperm injection, thyroid autoimmunity, vitamin D, early pregnancy outcomes

## Abstract

This prospective cohort study aimed to determine the impact of thyroid autoimmunity and total 25-hydroxyvitamin D concentration on early pregnancy outcomes in women undergoing *in vitro* fertilization/intracytoplasmic sperm injection who had intact thyroid function. The study included 1,297 women who underwent *in vitro* fertilization/intracytoplasmic sperm injection cycles, although only 588 patients received fresh embryo transfer. The study endpoints were clinical pregnancy, ongoing pregnancy, ectopic pregnancy, and early miscarriage rates. Our study found that the total 25-hydroxyvitamin D serum concentrations (*P<*0.001) and anti-Mullerian hormone levels (*P=*0.019) were lower among patients in the TAI group (n=518) than among those in the non-TAI group (n=779). Additionally, the study population in each group was divided into three subgroups according to the total vitamin D status based on clinical practice guidelines (deficient, <20 ng/mL; insufficient, 21–29 ng/mL; and sufficient, ≥30 ng/mL), TAI group: sufficient, n=144; insufficient, n=187; and deficient, n=187; non-TAI group: sufficient, n=329; insufficient, n=318, and deficient, n=133. In the TAI group, the number of good-quality embryos decreased in patients with vitamin D deficiency (*P=*0.007). Logistic regression analysis indicated that aging prevented women from achieving clinical (*P=*0.024) and ongoing pregnancy (*P=*0.026). The current findings suggest that patients with TAI had reduced serum vitamin D concentration. Furthermore, in the TAI group, the number of good-quality embryos decreased in patients with vitamin D deficiency. Finally, aging adversely impacted achieving clinical and ongoing pregnancy.

## 1 Introduction

*In vitro* fertilization (IVF) treatment of infertile couples has helped birth more than 8 million newborns worldwide. Although stimulation protocols and laboratory techniques have improved, the live birth rate per cycle remains between 19% and 22%. After years of practice, clinicians have achieved high treatment success rates (1).

Almost all aspects of biological activity can be correlated with vitamin D, a prohormone first characterized as a vitamin in the 20th century (2). The active metabolite of vitamin D, 1,25(OH)2 vitamin D, is crucial for regulating calcium and phosphate homeostasis and cell proliferation and differentiation. Moreover, the immune and nervous systems and cardiovascular and neurodegenerative diseases can be influenced in delicate ways by vitamin D. The fact that the vitamin D receptor could be detected in several tissues, including the ovary, endometrium, decidua, and placenta, as well as in fallopian epithelial cells (3), indicates that vitamin D may also be vital for the female reproductive system (4).

Thyroid autoimmunity (TAI) is the leading cause of primary hypothyroidism. When damaged by thyroid autoantibodies, thyroid tissue may ultimately be destroyed and lose the ability to maintain normal function (5). Studies have reported that patients with TAI present with decreased 25-hydroxyvitamin D [25(OH)D] levels (6, 7); after vitamin D supplementation, the titer of thyroid autoantibodies in patients with TAI and poor vitamin status decreased significantly (8). The relationship between TAI and the female reproductive system is also noteworthy. Reportedly, the miscarriage rates were higher in euthyroid women who tested positive for thyroid (thyroid peroxidase and thyroglobulin) antibodies in the first trimester of pregnancy (9). Additionally, the number of oocytes retrieved and birth weight in twin pregnancies decreased in patients with TAI (10). Moreover, TAI tends to be associated with IVF failure (11, 12) and various gynecological issues, including unexplained infertility (12, 13).

This study aimed to provide clear clues about the effects of TAI and vitamin D in patients with infertility undergoing IVF who had normal thyroid function.

## 2 Materials and methods

### 2.1 Study design and setting

This was a prospective, single-center cohort study. Infertile women undergoing IVF/intracytoplasmic sperm injection (ICSI) cycles between April 2021 and December 2021 were referred to the Reproductive Center of Peking University Third Hospital for IVF/ICSI treatment. The local ethics committee approved the study protocol (registration no. M2021189).

### 2.2 Participants

A total of 1,297 female patients who underwent IVF/ICSI cycles were enrolled in this study. The inclusion criteria were as follows: 1) age 20–40 years, 2) fresh embryo transfer (ET) recipients, 3) male or tubal factor infertility, and 4) normal thyroid function (defined as a TSH level within the reference range of 0.50–4.78 mIU/L). Patients with any of the following issues were excluded: 1) reproductive endocrinology disorders leading to infertility, including polycystic ovary syndrome, endometriosis, premature ovarian failure, hyperprolactinemia, and diminished ovarian reserve; 2) previous thyroidectomy; 3) autoimmune disorder complications; 4) cardiopulmonary, liver, or kidney diseases; 5) vitamin D supplementation; 6) IVF failure over three times; 7) pelvic/intrauterine adhesion or untreated hydrosalpinx; 8) recurrent abortion; 9) uterine malformation; or 10) uterine myoma (multiple, submucous, or intramural myoma >4 cm).

All the patients underwent IVF/ICSI cycles. The treatment protocols are described in the **Supplementary Materials and Methods**.

### 2.3 Study endpoints

The primary outcome was clinical pregnancy, defined as a gestational sac observed on ultrasonography 4 weeks after ET.

Secondary outcomes were as follows: 1) ongoing pregnancy, 2) ectopic pregnancy, and 3) early pregnancy loss. Ongoing pregnancy was defined as a gestational sac observed on ultrasonography 10 weeks after ET. Ectopic pregnancy was diagnosed using ultrasonography, intraoperatively, or histopathologically. Early pregnancy loss was defined as spontaneous abortion of intrauterine pregnancy within 12 weeks of gestation.

### 2.4 Laboratory testing

Blood samples for thyroid hormone testing were collected within 6 months before the initiation of controlled ovarian hyperstimulation (COS). The total serum 25(OH)D concentration was measured on the second day of the menstrual cycle. A fully automatic chemiluminescence immunoassay analyzer (ADVIA Centaur XP, Siemens Healthcare Diagnostics) was used to measure the serum free thyroxine (FT4), total thyroxin (TT4), thyroid-stimulating hormone (TSH), thyroid peroxidase antibody (TPOAb), and thyroglobulin antibody (TgAb) levels. Serum vitamin D levels were measured using a fully automatic chemiluminescence immunoassay analyzer (Elecsys Vitamin D total, Roche Diagnostics GmbH, Pleasanton, CA, United States of America). The reference values for TSH and FT4 were 0.55–4.78 µIU/mL and 0.89–1.80 ng/dL, respectively. TPOAb or TGAb levels <60 IU/mL were defined as negative.

### 2.5 Categorization

After being divided into two groups according to their thyroid antibody positivity, patients in both groups were also divided into three subgroups according to their vitamin D status, defined in the American Endocrine Society’s clinical practice guidelines as follows (14): deficient, <20 ng/mL (50 nmol/L); insufficient, 21–29 ng/mL (52.5–72.5 nmol/L); and sufficient, ≥30 ng/mL (75 nmol/L).

### 2.6 Statistical analyses

Categorical data are presented as the number of cases (percentage). Continuous data are presented as the mean (standard deviation) for normally distributed data and as the median (interquartile range) for non-normally distributed data. Continuous variables without normal distributions were compared using the Mann–Whitney U test. Student’s t-test/one-way analysis of variance and the chi-square test were used to compare continuous and categorical variables, respectively. A logistic regression model was used to analyze the association between early pregnancy outcomes and relevant factors. Statistical significance was defined as a two-sided P value <0.05. Statistical analyses were performed using SPSS version 24.0.

## 3 Results

A total of 1,297 participants who met the selection criteria were recruited to initiate the IVF/ICSI cycle. After removing 709 cycles cancelled before ET, 588 patients received fresh ET and continued to be observed for early pregnancy outcomes (clinical pregnancy, ongoing pregnancy, ectopic pregnancy, and early miscarriages). The characteristics of the study population are summarized in **Table 1**. The total 25(OH)D serum concentration (*P<*0.001) and anti-Mullerian hormone (AMH) (*P=*0.019) levels were higher in patients in the non-TAI group than in those in the TAI group. Furthermore, the FT4 (*P=*0.007), TT4 (*P=*0.001), and TSH (*P=*0.001) levels were slightly elevated in the TAI group. Vitamin D status was significantly different between the two groups (*P<*0.001); vitamin D deficiency was prevalent in 36.1% of the TAI group and 17.1% of the non-TAI group.

**Table 1:** Baseline characteristics of patients.

Characteristics	TAI (n=518)	Non-TAI (n=779)	P value
Age, median (IQR), y	33 (30–35)	32 (30–35)	0.072
Body mass index, median (IQR), kg/m^2^	22.1 (20.1–25.0)	22.0 (20.2–25.0)	0.711
Basal FSH, median (IQR), mIU/mL^a^	6.10 (5.08–7.31)	6.08 (4.97–7.38)	0.613
AMH, median (IQR), ng/mL	2.59 (1.66–4.08)	2.87 (1.80–4.49)	0.019
FT4, median (IQR), ng/dL	1.24 (1.14–1.35)	1.22 (1.12–1.33)	0.007
TT4, median (IQR), μg/dL	8.00 (7.00–9.20)	7.70 (6.90–8.65)	0.001
TSH, median (IQR), mIU/L	2.15 (1.54–2.98)	1.97 (1.41–2.63)	0.001
Serum 25(OH) level, median (IQR), ng/mL	21.7 (16.1–30.4)	28.8 (24.0–38.0)	<0.001
Serum 25(OH) status, No. (%)			<0.001
Sufficient	144 (27.8)	329 (42.2)	
Insufficient	187 (36.1)	317 (40.7)	
Deficient	187 (36.1)	133 (17.1)	
Antral follicle count in both ovaries, median (IQR)	12.0 (8.0–15.0)	12.0 (8.0–16.0)	0.867
No. of oocytes retrieved per cycle, median (IQR)	10.0 (6.0–16.0)	10.0 (6.0–16.0)	0.269
Fertilization rate, median (IQR), %	75.0 (55.6–90.0)	75.0 (55.6–90.0)	0.124
No. of good-quality embryos per cycle, median (IQR)^b^	4.0 (2.0–7.0)	4.0 (2.0–7.0)	0.589

FSH, follicle-stimulating hormone; AMH, anti-Mullerian hormone; FT4, free thyroxine; TT4, total thyroxine; IQR, interquartile range; TAI, thyroid autoimmunity; TSH, thyroid-stimulating hormone.

a Testing for basal FSH was performed on the second day of the menstrual cycle.

b Embryos were evaluated on the third day after fertilization. Good-quality embryos were developed from two pronuclei zygotes and met the following criteria: (1) had more than five blastomeres; (2) the size difference was <20%; and (3) fragmentation was <50%.

Women in each group were divided into three subgroups based on their 25(OH)D status. The analyses of baseline parameters according to 25(OH)D status are presented in **Table 2**. All participants with better vitamin D status tended to be older (TAI, *P=*0.024; non-TAI, 0.042) and had lower TT4 levels (*P<*0.001). In the TAI group, the TT4 level (*P=*0.015) and number of good-quality embryos (*P=*0.007) remained high among patients with better vitamin D status. In the non-TAI group, body mass index (BMI) was inversely correlated with vitamin D status (*P=*0.018).

**Table 2:** Baseline characteristics of patients according to vitamin D status and TAI/non-TAI.

Characteristics	TAI (n=518)				Non-TAI (n=779)			
	Sufficient (≥30 ng/mL) (n=144)	Insufficient (20–30 ng/mL) (n=187)	Deficient (<20 ng/mL) (n=187)	P value	Sufficient (≥30 ng/mL) (n=329)	Insufficient (20–30 ng/mL) (n=317)	Deficient (<20 ng/mL) (n=133)	P value
Age, median (IQR), y	33 (31–36)	32 (30–35)	32 (30–35)	0.024	33 (30–35)	32 (29–35)	31 (29–34)	0.042
Body mass index, median (IQR), kg/m^2^	21.9 (20.3–23.9)	22.6 (20.4–25.4)	22.0 (19.6–24.6)	0.085	21.5 (19.9–25.4)	21.8 (20.0–24.2)	22.7 (20.5–25.2)	0.018
Basal FSH, median (IQR), mIU/mL^a^	6.21 (5.25–7.41)	6.07 (5.17–7.35)	6.01 (4.96–7.13)	0.328	6.09 (4.98–7.33)	6.07 (4.91–7.50)	6.10 (5.14–7.28)	0.950
AMH, median (IQR), ng/mL	2.66 (1.68–4.03)	2.48 (1.65–3.99)	2.79 (1.66–4.28)	0.487	2.77 (1.77–4.45)	2.92 (1.78–4.71)	3.05 (2.06–4.29)	0.506
FT4, median (IQR), ng/dL	1.22 (1.14–1.32)	1.26 (1.17–1.37)	1.26 (1.13–1.36)	0.169	1.23 (1.12–1.32)	1.22 (1.12–1.34)	1.23 (1.13–1.33)	0.939
TT4, median (IQR), μg/dL	7.80 (6.80–9.10)	7.90 (6.80–9.00)	8.30 (7.30–9,40)	0.015	7.60 (6.80–8.70)	7.70 (6.85–8.50)	7.90 (7.05–9.00)	0.159
TSH, median (IQR), mIU/L	2.04 (1.50–2.79)	2.22 (1.61–2.98)	2.14 (1.48–3.17)	0.498	1.91 (1.42–2.68)	1.98 (1.38–2.55)	2.04 (1.50–2.60)	0.943
Antral follicle count in both ovaries, median (IQR)	11.0 (8.0–14.0)	12.0 (9.0–16.0)	12.0 (8.0–16.0)	0.091	12.0 (8.0–16.0)	12.0 (8.0–16.0)	11.0 (8.0–14.5)	0.386
No. of oocytes retrieved per cycle, median (IQR)	10.0 (6.0–15.0)	11.0 (6.0–15.0)	11.0 (7.0–15.0)	0.578	10.0 (6.0–17.0)	10.0 (6.0–16.0)	9.0 (6.0–14.0)	0.272
Fertilization rate, median (IQR), %	71.4 (50.0–88.9)	70.8 (50.0–86.7)	75.0 (57.1–87.5)	0.709	73.3 (56.1–88.9)	75.0 (56.8–92.3)	71.4 (50.0–87.5)	0.526
No. of good-quality embryos per cycle, median (IQR)^b^	4.0 (2.0–7.0)	4.0 (2.0–7.0)	3.0 (1.0–5.0)	0.007	4.0 (2.0–7.0)	4.0 (2.0–7.0)	4.0 (2.0–6.0)	0.797

FSH, follicle-stimulating hormone; AMH, anti-Mullerian hormone; FT4, free thyroxine; TT4, total thyroxine; IQR, interquartile range; TAI, thyroid autoimmunity; TSH, thyroid-stimulating hormone.

a Testing for basal FSH was performed on the second day of the menstrual cycle.

b Embryos were evaluated on the third day after fertilization. Good-quality embryos were all developed from two pronuclei zygotes and met the following criteria: 1) more than five blastomeres; 2) size difference <20%; and 3) fragmentation <50%.

Furthermore, the chi-square test showed that patients in the TAI group presented with increased early miscarriage rate (*P*=0.034). When participants in each group were divided into three subgroups according to their vitamin D status, no significant differences were observed when comparing all the TAI and non-TAI study primary and secondary outcomes (**Table 3**).

**Table 3:** Comparison of early pregnancy outcomes according to TAI/non-TAI and total 25(OH)D status in 588 patients who underwent fresh embryo transfer.

		Clinical pregnancy	Ongoing pregnancy	Ectopic pregnancy	Early miscarriage
TAI		43.1% (100/232)	32.3% (75/232)	1.7% (4/232)	25% (25/100)
Non-TAI		46.3% (165/356)	39.6% (141/356)	2.2% (8/356)	14.5% (24/165)
P value		0.440	0.074	0.661	0.034
TAI	Sufficient (25(OH)D >30 ng/mL)	47.8 (32/67)	31.8 (28/67)	0.0 (0/67)	12.5 (4/32)
	Insufficiency (25(OH)D 20–30 ng/mL)	39.3 (35/89)	28.1 (25/89)	2.2 (2/89)	28.6 (10/35)
	Deficiency (25(OH)D <20 ng/mL)	43.4 (33/76)	28.9 (22/76)	2.6 (2/76)	33.3 (11/33)
	P value	0.573	0.144	0.430	0.127
Non-TAI	Sufficient (25(OH)D >30 ng/mL)	46.3 (81/175)	40.0 (70/175)	2.9 (5/175)	13.6 (11/81)
	Insufficiency (25(OH)D 20–30 ng/mL)	46.4 (58/125)	39.2 (49/125)	2.4 (3/2.4)	15.5 (9/58)
	Deficiency (25(OH)D <20 ng/mL)	46.4 (26/56)	39.3 (22/56)	0.0 (0/56)	15.4 (4/26)
	P value	0.982	0.989	0.450	0.942

Data are presented as % (n/N). Clinical pregnancy, presence of a gestational sac with fetal heartbeat on transvaginal ultrasound 4 weeks after transfer; ongoing pregnancy, presence of gestational sac with fetal heartbeat on transvaginal ultrasound at 11 weeks of gestation; early miscarriage, spontaneous loss of an intrauterine pregnancy at ≤12 weeks of gestation; and (OH)D, 25-hydroxyvitamin D.

Logistic regression analysis for clinical pregnancy, ongoing pregnancy, and early miscarriage considering age, BMI, AMH, serum vitamin D status, infertility type, FT4, TT4, TSH, TAI/non-TAI, protocol of COS, antral follicle count, number of retrieved oocytes and good-quality embryos, and embryo culture duration (day 3 or 5) only indicated that aging prevented women from achieving clinical pregnancy (0.948; 95% confidence interval [CI], 0.905–0.993; *P=*0.024) and ongoing pregnancy (0.946; 95% CI, 0.901–0.993; *P=*0.026) (**Table 4**). TAI, vitamin D status and other relevant factors stated above didn’t influence all early pregnancy outcomes. After considering the interactions between total serum 25(OH)D status and TAI/non-TAI as confounding factors, no significant difference was observed between the interactions and any of the study endpoints (*P >*0.05) (**Table 4**).

**Table 4:** Logistic regression analysis of clinical pregnancy, ongoing pregnancy, and early miscarriage according to demographic and metabolic factors.

	Clinical pregnancy				Ongoing pregnancy				Early miscarriage			
	β	Standard error	OR (95% CI)	P value	β	Standard error	OR (95% CI)	P value	β	Standard error	OR (95% CI)	P value
Age (y)	-0.053	0.024	0.948 (0.905–0.993)	0.024	-0.058	0.024	0.943 (0.899-0.989)	0.017	0.050	0.051	1.051 (0.952-1.161)	0.326
BMI (kg/m^2^)	-0.004	0.023	0.996 (0.952–1.041)	0.855	-0.003	0.023	0.997 (0.953-1.043)	0.896	-0.039	0.048	0.962 (0.875-1.057)	0.418
AMH (ng/mL)	0.043	0.043	1.044 (0.960–1.135)	0.314	0.024	0.043	1.025 (0.942-1.115)	0.574	0.038	0.086	1.039 (0.878-1.230)	0.655
Total 25(OH)D status												
Sufficient			1				1				1	
Insufficient	-0.038	0.244	0.963 (0.597–1.553)	0.877	-0.281	0.203	0.755 (0.508-1.124)	0.166	0.229	0.501	1.258 (0.471-3.356)	0.647
Deficient	-0.086	0.319	0.918 (0.491–1.716)	0.787	-0.321	0.242	0.725 (0.452-1.165)	0.184	0.167	0.657	1.182 (0.326-4.284)	0.800
FT4 (ng/dL)	-0.080	0.272	0.923 (0.542–1.572)	0.768	-0.135	0.313	0.873 (0.473-1.614)	0.666	-0.174	0.953	0.840 (0.130-5.438)	0.855
TT4	0.024	0.052	1.024 (0.926–1.133)	0.643	-0.036	0.053	0.964 (0.869-1.070)	0.496	0.182	0.102	1.199 (0.982-1.465)	0.075
TSH (mIU/L)	-0.108	0.086	0.897 (0.757–1.063)	0.210	-0.137	0.091	0.872 (0.730-1.041)	0.130	0.202	0.168	1.224 (0.880-1.702)	0.229
Non-TAI			1				1				1	
TAI	0.212	0.299	1.236 (0.687–2.223)	0.479	-0.149	0.189	0.862 (0.595-1.248)	0.431	-0.290	0.640	0.748 (0.213-2.625)	0.651
Antral follicle count	0.019	0.017	1.019 (0.986–1.053)	0.267	0.017	0.017	1.017 (0.984-1.052)	0.317	-0.006	0.034	0.994 (0.930-1.062)	0.864
No. of oocytes retrieved	0.012	0.016	1.012 (0.981–1.044)	0.460	0.010	0.013	1.010 (0.984-1.036)	0.474	-0.020	0.030	0.980 (0.924-1.041)	0.514
No. of good-quality embryos	0.033	0.043	1.034 (0.951–1.124)	0.439	-0.020	0.028	0.981 (0.929-1.035)	0.476	0.046	0.056	1.047 (0.937-1.170)	0.416
Embryo culture duration^a^												
Day 3 (n=563)			1				1				1	
Day 5 (n=25)	-0.401	0.439	0.670 (0.283–1.583)	0.361	-0.367	0.464	0.692 (0.279-1.721)	0.429	0.193	0.847	1.213 (0.231-6.376)	0.820
Non-TAI by vitamin D sufficient			1				1				1	
TAI by vitamin D insufficient	-0.348	0.413	0.706 (0.314-1.587)	0.400	-0.622	0.429	0.537 (0.232-1.244)	0.147	0.983	0.839	2.674 (0.516-13.845)	0.241
TAI by vitamin D deficient	-0.312	0.469	0.732 (0.292-1.835)	0.506	-0.563	0.486	0.569 (0.220-1.475)	0.246	0.928	0.940	2.531 (0.401-15.959)	0.323

Clinical pregnancy, presence of gestational sac with fetal heartbeat on transvaginal ultrasound 4 weeks after embryo transfer; ongoing pregnancy, presence of gestational sac with fetal heartbeat on transvaginal ultrasound at 11 weeks of gestation; early miscarriage, spontaneous loss of intrauterine pregnancy at ≤12 weeks of gestation. BMI, body mass index; AMH, anti-Mullerian hormone; FT4, free thyroxine; TT4, total thyroxine; TAI, thyroid autoimmunity; TSH, thyroid-stimulating hormone; 25(OH)D, 25-hydroxyvitamin D.

Binary logistic regression model also incorporated infertility type and protocol of COS as relevant factors.

a Embryos were transferred either on day 3 or day 5.

## 4 Discussion

Our study’s strengths include being the first prospective cohort study to explore the association among TAI, serum vitamin D level, and early pregnancy outcomes in patients undergoing IVF treatment. Additionally, the sample size was relatively large leading to better-quality results. We found that TAI patients tended to have reduced serum 25(OH)D concentrations. In the TAI group, the number of good-quality embryos decreased in patients with vitamin D deficiency. Furthermore, aging adversely impacted achieving clinical and ongoing pregnancy. Considering the prevalence and potential fertility-damaging impact of TAI, testing for thyroid function and thyroid antibody is recommended for infertile patients in China. A study conducted by Chao et al. (6), involving 5,230 Chinese participants, showed that vitamin D deficiency is prevalent in over 70% in both the Hashimoto’s thyroiditis (HT) and non-HT groups within the Chinese population. These data combined with the findings of our study indicate that treatment with vitamin D may help to improve IVF/ICSI outcomes; large, prospective, randomized controlled studies are warranted to confirm these findings.

Furthermore, there is ambiguous predictability between vitamin D and assisted reproductive technology (ART) outcomes. Pregnancy loss is linked to vitamin D deficiency in women undergoing ART (15). According to a recent Cochrane analysis (16) and another review (17), vitamin D supplementation in pregnant women may reduce the risk of preeclampsia, low birth weight, preterm birth, and wheezing in offspring at 3 years of age. An association between vitamin D deficiency and BMI has also been reported (18). This meta-analysis showed that each 10% increase in BMI leads to a 4% decrease in the 25(OH)D concentration; among obese populations receiving ART, vitamin D deficiency could contribute to a higher miscarriage rate. Similarly, our study showed that the vitamin D concentration was inversely correlated with BMI in the non-TAI group. Several studies investigating the relationship between female vitamin D status and IVF outcomes concluded that higher vitamin D levels lead to a higher clinical pregnancy rate (CPR) (19–22). However, negative results in several studies suggest no link between vitamin D levels and IVF outcomes (23–25). Anifandis et al. (26) found that higher values of vitamin D were associated with a lower possibility of achieving clinical pregnancy. Recent research (27) has shown that women with a sufficient level of vitamin D had higher ongoing pregnancy rate than those with a deficiency or insufficient level of vitamin D. Our study only found that the number of good-quality embryos decreased in patients with vitamin D deficiency in the TAI group, following the negative findings considering early pregnancy outcomes presented above in both TAI and non-TAI groups with different serum vitamin D status. However, we have ruled out potentially vitamin D-relevant diseases leading to infertility, such as polycystic ovary syndrome, endometriosis, premature ovarian failure, and diminished ovarian reserve. The difference in the study participants may have accounted for the discrepancy in the results, noting that participants with other causes of infertility need to be further studied and explained.

TAI is a chronic autoimmune phenomenon. In COS and early pregnancy, the thyroid gland may fail to respond to aggregated demand after experiencing a subtle deficiency caused by thyroid autoantibodies that may compromise the establishment of immune tolerance during pregnancy (9). However, the association between TAI and IVF outcomes remains controversial. A recent study that aimed to further clarify the association between vitamin D deficiency and HT featuring TAI recruited 5,230 healthy subjects who underwent health examinations. The mean age was 48.95 ± 9.06 years, and 60.1% were male individuals. The researchers discovered that HT patients present with reduced 25(OH)D levels (6), which is consistent with our findings that the total 25(OH)D serum concentration was lower in patients in the TAI group than in those in the TAI group (21.7 [16.1–30.4] ng/mL and 28.8 [24.0–38.0] ng/mL in the TAI and non-TAI groups, respectively), with vitamin D deficiency prevalent in 36.1% of the TAI group and 17.1% of the non-TAI group. Several studies have found that women undergoing TAI may have adverse outcomes. After enrolling 1,556 infertile patients who received their first IVF/ICSI treatment and achieved fresh ET at the Reproductive Center of Peking University Third Hospital, a large-scale retrospective cohort study revealed that the presence of TAI could decrease the number of oocytes retrieved (10). After enrolling 122 patients aged 20–40 years who received IVF/ICSI treatment, a cross-sectional study focusing on the follicular microenvironment of patients with TAI showed that levels of three chemokines (CXCL9/10/11), one cytokine (IFN-γ), and the percentage of CXCR3+ T lymphocyte were elevated, suggesting an occurrence of immunological imbalance (28). It was also reported that TAI is linked to reduced CPR (29), decreased birth or delivery rate (10, 29, 30), and increased miscarriage rate (30, 31); however, evidence from other studies does not support the results mentioned above (32, 33). We found that patients in TAI group presented with increased early miscarriage rate using the chi-square test, but the Logistic regression analysis didn’t prove that TAI contributed to early miscarriage. Besides, the large-scale retrospective cohort study with 1,556 participants at the Reproductive Center of Peking University Third Hospital demonstrated that the early miscarriage rate among patients with TAI wasn’t increased (10), and the sample size of patients with early miscarriage in this study was bigger than ours (100 vs. 49), requiring further prospective study with larger sample size to confirm the impact of TAI on IVF/ICSI outcomes and provide scientific basis for clinical practice. Our study also demonstrated that patients with TAI with vitamin D deficiency tended to have a lower number of good-quality embryos. In this case, vitamin D and TAI likely served as interactive factors that may have influenced the results. This concern is based on the fact that no significant difference between the number of good-quality embryos was found among patients after being divided into two groups according to TAI positivity or into three groups according to vitamin D status. In addition, the lack of studies investigating the interaction between TAI and vitamin D on IVF outcomes has made this a poorly worded question. However, our study found that AMH levels were lower; FT4, TT4, and TSH levels were elevated; and TT4 levels were inversely correlated with the 25(OH)D concentration in the TAI group, suggesting that vitamin D and the thyroid play a possible role in affecting follicle development in TAI patients. A previous study reported that serum TSH concentrations were negatively correlated with IVF oocyte maturation and fertilization rate (34).

Our study also revealed that patients in the TAI group had lower AMH levels. After being secreted by granulosa cells (GCs), AMH regulates the development of early follicular and thus serves as a marker of the ovarian reserve (35). However, studies on AMH levels mostly reported the correlation between vitamin D and AMH levels. Wojtusik et al. (36) found that vitamin D treatment decreased AMH expression in GCs in small follicles, and larger follicles expressed higher level of vitamin D receptor than small follicles. Bednarska-Czerwinska et al. (37) reported that AMH levels were negatively correlated with follicular fluid (FF) vitamin D levels. Similarly, Merhi and colleagues (38) described AMH and AMH receptor (AMHR) gene expression in GCs as being negatively correlated with FF vitamin D among reproductive-aged females. On the other hand, after recruiting 388 premenopausal women in their cross-sectional study, Merhi and colleagues described AMH level as being positively correlated with blood vitamin D levels in women over the age of 40 years (39). However, there are few studies focusing on the correlation between TAI and AMH level and the effect of TAI on AMH expression in GCs.

After being divided into three subgroups, older patients in both the TAI and non-TAI groups presented with sufficient vitamin D levels. One possible explanation is that delayed child wishes often meet with age-related fertility loss, which starts at around 25–30 years of age (40). This delayed child wish pushed women to the reproductive center for fertility assessment, thus introducing them to more opportunities to be tested if they are TAI positive or vitamin D sufficient. Another likely factor is the lack of sun exposure. Sunlight exposure remains a major source of vitamin D (41). Protection from the sun through clothing and glass absorbing all of the ultraviolet B radiation prevents vitamin D production during sun exposure, which could be a matter of concern in China.

This study has some limitations. First, it was a single-center study. However, the quality control checks of the IVF-ET process and laboratory measurements were performed better in a single center than in multiple centers. In addition, our center performs >18,000 cycles of IVF-ET per year; the fact that all patients were scattered across the country makes the study’s population geographically representative. Second, lack of data concerning daylight hours, seasonal factors, outdoor activity conditions, and iodine nutritional status. Third, Han Chinese women only with male or tubal infertility made up the main study population, asking the evaluation of other races or populations with other causes of infertility to ensure the applicability of our findings.

## 5 Conclusion

This study revealed that patients with TAI tend to have reduced serum 25(OH)D concentration. Additionally, in the TAI group, the number of good-quality embryos decreased in patients with poor vitamin D status. Lastly, among women who underwent fresh ET, aging adversely impacted achieving clinical and ongoing pregnancy.

## Data availability statement

The raw data supporting the conclusions of this article will be made available by the authors, without undue reservation.

## Ethics statement

The studies involving human participants were reviewed and approved by Reproductive Center of Peking University Third Hospital for IVF/ICSI treatment. The local ethics committee approved the study protocol (registration no. M2021189). The patients/participants provided their written informed consent to participate in this study.

## Author contributions

YL, ZH, NH, and HC conceptualized the study, and all the authors contributed to the research discussion. YL, ZH, YW, LZ, and RL took part in patient follow-up and performed data analysis. YL wrote the initial draft of the paper, and all authors contributed to manuscript revision. All authors contributed to the article and approved the submitted version.
