# Supplementary material for: Impact of thyroid autoimmunity and vitamin D on in vitro fertilization/intracytoplasmic sperm injection outcomes among women with normal thyroid function
Source: Front Endocrinol (Lausanne). 2023 May 8;14:1098975. doi: 10.3389/fendo.2023.1098975 (PMC10200944; doi:10.3389/fendo.2023.1098975)
Supplement: Supplementary file 1 [file Table_1.doc]

**Supplementary Material**

All patients underwent a standardised, controlled ovarian stimulation regimen, oocyte retrieval, and fertilisation, followed by fresh embryo transfer in in vitro fertilization (IVF)/intracytoplasmic sperm injection (ICSI) cycles. The protocols are as follows. 1) Patients treated with the antagonist protocol: recombinant gonadotropins and gonadotropin-releasing hormone (GnRH) antagonist were initiated on the 2nd day of the menstrual cycle and the day when at least one follicle reached 12 mm in diameter respectively, and the treatment was repeated until the day when human chorionic gonadotropin (HCG) was administered. 2) Patients treated with the short-term protocol: short-acting GnRH agonist and recombinant gonadotropins were injected for ovarian stimulation. 3) Patients underwent the ultralong-term and long-term protocols: recombinant gonadotropins were injected for ovarian stimulation after downregulation was achieved using a long-acting GnRH agonist.

The individualised dose of gonadotropins was decided based on the patient’s age, BMI, and anti-Müllerian hormone levels. Recombinant HCG 250 μg (Eiser, Serono, Germany) was administered to trigger oocyte maturation when at least two follicles reached 18 mm in diameter. Oocyte retrieval was performed 34–36 hours after HCG administration. Insemination was performed at 4–6 hours after oocyte retrieval using a routine IVF method or ICSI injection according to the sperm quality. Up to two day-3 embryos or blastocysts were transferred 3 or 5 days after oocyte retrieval.
